# Supplementary material for: Improving prediction of cervical cancer using KNN imputer and multi-model ensemble learning
Source: PLoS One. 2024 Jan 3;19(1):e0295632. doi: 10.1371/journal.pone.0295632 (PMC10763959; doi:10.1371/journal.pone.0295632)
Supplement: Attachment — Submitted filename: Response_letter_Plos_One_Turki_Aljrees.pdf [file pone.0295632.s001.pdf]

Manuscript ID: PONE-D-23-24373

Article title: Improving Prediction of Cervical Cancer Using KNN Imputer and Multi-Model Ensemble Learning

October 5, 2023

Dear Academic Editor,

First of all, we would like to extend our sincere gratitude to Editor in Chief, Academic Editor, for allowing a re-submission of our manuscript, with an opportunity to address the reviewer's comments. We greatly appreciate the Reviewers' time spent reviewing the paper and assisting in its improvement. These comments are very helpful for revising and improving the paper, as well as the important guiding significance to the other research. We have studied the comments very carefully and made the corrections that we hope to meet with the final approval. We have done our best to modify the paper to reflect all the comments made by anonymous reviewers. These changes are fully addressed in this reply letter.

Below, we provide point-to-point responses explaining how we have addressed each of the Reviewer's comments. For clarity, our responses have been written in blue color. whenever we copy text from the manuscript, it is given in red color.

REVIEWER 1

Concern 1

Introduction section need revision, it is recommended to present one idea in one paragraph. As in paragraph one, the author introduces cervical cancer and its statistics, which are again repeated in paragraph 3. In addition, provide some information about some common methods used to detect cervical cancer survival rate.

Response: We are thankful for your insightful comment. Respected reviewer, We have revised the introduction section.

Concern 2

Reconsider the Related work section to incorporate recently published works for cervical cancer survival prediction.

Response: The authors are grateful for your valuable suggestions. We have added recently published works for cervical cancer survival prediction in the related work section.

Concern 3

Clearly mention the research gap.

Response: We are thankful for your insightful comment. Respected reviewer, We have added a research gap in the introduction section with the following lines:

The research in cervical cancer detection aims to address a critical gap in existing methods, primarily related

to the handling of missing data in machine learning approaches. While acknowledging the significance of early and accurate cervical cancer detection, the paper highlights the limitations of traditional methods and emphasizes the potential of machine learning. The research gap is identified as the lack of comprehensive studies and robust methodologies for handling missing data in cervical cancer datasets.

Concern 4

Highlight the motivation and rationale of the study.

Response: The authors highly appreciate your valuable suggestions. Respected reviewer, we have added the motivation and rationale of the study with following lines:
Cervical cancer is a major public health concern, especially in regions with high incidence rates. Existing screening methods have limitations, and there is an urgent need to improve cervical cancer diagnosis. This study explores the potential of EHRs and machine learning to develop more accurate and efficient diagnostic tools. Unfortunately, there are many countries without reliable screening techniques for finding this kind of cancer. Existing data sets are not suitable for machine learning models because they may contain missing or null values, which have a significant influence on the effectiveness of models.

Concern 5

Improve images to make them more clear.

Response: Respected reviewer, We have improved the quality of the images.

Concern 6

Empirical analysis of the proposed methodology is missing.

Response: Respected reviewer, Empirical analysis involves experimental results, performance metrics, and comparisons with existing methods. All of these are provided in the Results and Discussion section.

Concern 7

Provide ablation study to show the significance of each part of the proposed methodology.
Response: Respected reviewer, the novelty of this research work lies in the imputation of missing values with the KNN imputing technique. We have shows the effect of missing values in three aspects with KNN imputation, removing missing values, and with PCA imputation. This itself reflects the ablation study of this research work proposed methodology that imputation of missing values with KNN does the key role for accurate cervical cancer prediction.

Concern 8

Why PCA was used and how it only selected the important features.

Response: We are thankful for your insightful comment. Respected reviewer, we have added the complete procedure of PCA imputation in the paper.

PCA imputation techniques for filling in missing values in a dataset involve the following steps:

1. Data Preparation: Begin with a dataset that contains missing values. These missing values exist in more than one column or feature of the dataset.
2. Data Standardization: Standardize the dataset by centering it (subtracting the mean) and scaling it (dividing by the standard deviation). This step ensures that all features have similar scales, which is important for PCA.
3. PCA: Apply Principal Component Analysis to the dataset. PCA identifies the principal components

(linear combinations of the original features) that capture the most variance in the data. These principal components are ordered by their ability to explain variance, with the first component explaining the most variance, the second component explaining the second most, and so on.

4. Imputation: For missing values in the dataset, we use the information from the PCA-transformed data to impute those missing values. This typically involves projecting the data with missing values onto the PCA space and then back-transforming it to the original feature space. The imputed values are obtained by using the coordinates of the projected data on the principal components.

PCA imputation is based on the assumption that the variance in the data captures meaningful patterns, and it leverages this variance to estimate missing values. It can be a useful technique when dealing with datasets with missing values, especially when those missing values are missing at random like in this case.

Concern 9

Overall formatting needs reconsideration, at places text seems be unaligned, and indentation is not consistent.

Response: Respected Reviewer, no indentation is added in the latex class of PLoS One. it is by default no indent.

Concern 10

Number of grammatical and typo mistakes are present. Thoroughly revise the paper

Response: Respected reviewer, We have corrected the grammatical and typo mistakes in the manuscript.

REVIEWER 2

Concern 1

The abstract missed the novelty, Challenges, validation techniques, and some suggestion. Impact on social and Clinical.

Response: We are thankful for your insightful comment. Respected reviewer, We have revised the abstract. The updated one is:

Cervical cancer is a leading cause of women's mortality, emphasizing the need for early diagnosis and effective treatment. In line with the imperative of early intervention, the automated identification of cervical cancer has emerged as a promising avenue, leveraging machine learning techniques to enhance both the speed and accuracy of diagnosis. However, an inherent challenge in the development of these automated systems is the presence of missing values in the datasets commonly used for cervical cancer detection. Missing data can significantly impact the performance of machine learning models, potentially leading to inaccurate or unreliable results. This study addresses a critical challenge in automated cervical cancer identification—handling missing data in datasets. The study present a novel approach that combines three machine learning models into a stacked ensemble voting classifier, complemented by the use of a KNN Imputer to manage missing values. The proposed model achieves remarkable results with an accuracy of 0.9941, precision of 0.98, recall of 0.96, and an F1 score of 0.97. This study examines three distinct scenarios: one involving the deletion of missing values, another utilizing KNN imputation, and a third employing PCA for imputing missing values. This research has significant implications for the medical field, offering medical experts a powerful tool for more accurate cervical cancer therapy and enhancing the overall effectiveness of testing procedures. By addressing missing data challenges and achieving high accuracy, this work represents

a valuable contribution to cervical cancer detection, ultimately aiming to reduce the impact of this disease on women's health and healthcare systems.

Concern 2

The introduction section requires revision, with a suggestion to present one idea per paragraph. For instance, the author discusses cervical cancer and its statistics in both the first and third paragraphs. Furthermore, it would be beneficial to include details about common methods used for detecting cervical cancer survival rates.

Response: The authors are grateful for your valuable suggestions. Respected reviewer, We have revisited and enhanced the introduction section of the paper, discussing the common methods employed for the detection of cervical cancer survival rates.

Concern 3

Revisiting the Related Work section would be advantageous to incorporate recently published research on cervical cancer survival prediction.

Response: Respected reviewer, We have added recently published research on cervical cancer survival prediction in the related work section.

Concern 4

Clearly outlining the research gap would enhance the paper's clarity.

Response: The authors highly appreciate your valuable suggestions.Respected reviewer, We have added research gap in the introduction section with the following lines:
The research in cervical cancer detection aims to address a critical gap in existing methods, primarily related to the handling of missing data in machine learning approaches. While acknowledging the significance of early and accurate cervical cancer detection, the paper highlights the limitations of traditional methods and emphasizes the potential of machine learning. The research gap is identified as the lack of comprehensive studies and robust methodologies for handling missing data in cervical cancer datasets.

Concern 5

The proposed work is based on KNN/PCA which is state of the art algorithm, however, a lot of work exists on the proposed techniques. Please, mention your novelty in detail, rational, and impact of your techniques.

Response: Respected reviewer, The proposed work builds upon state-of-the-art algorithms, KNN and PCA, for cervical cancer detection. Its novelty lies in the integration of these techniques into a unified framework, the comprehensive handling of missing data, and rigorous empirical analysis. The rationale is to enhance accuracy and address the gap in missing data handling within cervical cancer datasets. The potential impact includes improved clinical outcomes and the advancement of healthcare analytics, making it a valuable contribution to the field.

Concern 6

Please, provide the detail ablation study in terms of results, and computational complexity.

Response: Respected reviewer, the novelty of this research work lies in the imputation of missing values with the KNN imputing technique. We have shows the effect of missing values in three aspects with KNN imputation, removing missing values, and with PCA imputation. This itself reflects the ablation study of this research work proposed methodology that imputation of missing values with KNN does the key role for accurate cervical cancer prediction.

Concern 7

Please, give limitation of previous work and critical research gap.

Response: We are thankful for your insightful comment. Respected reviewer, We have added a research gap in the introduction section with the following lines:
The research in cervical cancer detection aims to address a critical gap in existing methods, primarily related to the handling of missing data in machine learning approaches. While acknowledging the significance of early and accurate cervical cancer detection, the paper highlights the limitations of traditional methods and emphasizes the potential of machine learning. The research gap is identified as the lack of comprehensive studies and robust methodologies for handling missing data in cervical cancer datasets.

Concern 8

Please, provide the detail ablation study in terms of results, and computational complexity.

Response: Respected reviewer, the novelty of this research work lies in the imputation of missing values with the KNN imputing technique. We have shown the effect of missing values in three aspects with KNN imputation, removing missing values, and with PCA imputation. This itself reflects the ablation study of this research work proposed methodology that imputation of missing values with KNN does the key role for accurate cervical cancer prediction.

Concern 9

Emphasizing the motivation and rationale behind the study is important.

Response: Respected reviewer, We are motivated by the need for accurate early detection of cervical cancer, aiming to overcome limitations in traditional methods. Our rationale centers on the potential of integrating KNN and PCA within a novel framework to enhance diagnostic accuracy. Additionally, we address the crucial challenge of handling missing data in cervical cancer datasets. These motivations and rationales underpin our research, shaping its objectives and methodologies.
We have emphasized this point in the introduction section by highlighting the research gap.

Concern 10

Images could be improved to enhance their clarity and comprehension.

Response: Respected Reviewer, We have improved the quality of images.

Concern 11

The paper lacks empirical analysis of the proposed methodology.

Response: Respected reviewer, Empirical analysis involves experimental results, performance metrics, and comparisons with existing methods. All of these are provided in the Results and Discussion section.

Concern 12

The literature review can be strengthened by refereeing the articles

- Detection of tumour infiltrating lymphocytes in CD3 and CD8 stained histopathological images using a two-phase deep CNN.
- Brain tumor MRI Classification using a Novel Deep Residual and Regional CNN

- Coronavirus disease analysis using chest X-ray images and a novel deep convolutional neural network
- A New Deep Hybrid Boosted and Ensemble Learning-Based Brain Tumor Analysis Using MRI
- Attention-guided multi-scale deep object detection framework for lymphocyte analysis in IHC histological images
- Malaria parasite classification framework using a novel channel squeezed and boosted CNN

Response: Thank you very much for your insightful suggestions. Respected Reviewer, We have incorporated the previously mentioned works into the related work section and also cited them. The paragraph of the related work is:

In recent times, machine learning and deep learning models have emerged as valuable solutions for disease diagnosis like tumour infiltrating [7], brain tumour classification [8, 9], COVID-19 diagnosis [4], lymphocyte analysis [6], and Malarial parasite classification [3]. Researchers applied deep learning models to predict the survival rate of cervical cancer patients [1]. Authors applied deep learning models on the pap-smear dataset to classify cervical cancer [5]. Pretrained models like AlexNet, GoogleNet and ResNET are employed for feature extraction and machine learning models are used for classification using Pap smear dataset for cervical cancer prediction [2].

References

[1] Chi Chen, Yuye Cao, Weili Li, Zhenyu Liu, Ping Liu, Xin Tian, Caixia Sun, Wuliang Wang, Han Gao, Shan Kang, et al. The pathological risk score: A new deep learning-based signature for predicting survival in cervical cancer. *Cancer Medicine*, 12(2):1051–1063, 2023.

[2] Madhura Kalbhor, Swati Shinde, Hrushikesh Joshi, and Pankaj Wajire. Pap smear-based cervical cancer detection using hybrid deep learning and performance evaluation. *Computer Methods in Biomechanics and Biomedical Engineering: Imaging & Visualization*, pages 1–10, 2023.

[3] Saddam Hussain Khan, Najmus Saher Shah, Rabia Nuzhat, Abdul Majid, Hani Alquhayz, and Asifullah Khan. Malaria parasite classification framework using a novel channel squeezed and boosted cnn. *Microscopy*, 71(5):271–282, 2022.

[4] Saddam Hussain Khan, Anabia Sohail, Muhammad Mohsin Zafar, and Asifullah Khan. Coronavirus disease analysis using chest x-ray images and a novel deep convolutional neural network. *Photodiagnosis and Photodynamic Therapy*, 35:102473, 2021.

[5] Ishak Pacal and Serhat Kılıcarslan. Deep learning-based approaches for robust classification of cervical cancer. *Neural Computing and Applications*, 35(25):18813–18828, 2023.

[6] Zunaira Rauf, Anabia Sohail, Saddam Hussain Khan, Asifullah Khan, Jeonghwan Gwak, and Muhammad Maqbool. Attention-guided multi-scale deep object detection framework for lymphocyte analysis in ihc histological images. *Microscopy*, 72(1):27–42, 2023.

[7] Muhammad Mohsin Zafar, Zunaira Rauf, Anabia Sohail, Abdul Rehman Khan, Muhammad Obaidullah, Saddam Hussain Khan, Yeon Soo Lee, and Asifullah Khan. Detection of tumour infiltrating lymphocytes in cd3 and cd8 stained histopathological images using a two-phase deep cnn. *Photodiagnosis and Photodynamic Therapy*, 37:102676, 2022.

[8] Mirza Mumtaz Zahoor and Saddam Hussain Khan. Brain tumor mri classification using a novel deep residual and regional cnn. *arXiv preprint arXiv:2211.16571*, 2022.

[9] Mirza Mumtaz Zahoor, Shahzad Ahmad Qureshi, Sameena Bibi, Saddam Hussain Khan, Asifullah Khan, Usman Ghafoor, and Muhammad Raheel Bhutta. A new deep hybrid boosted and ensemble learning-based brain tumor analysis using mri. *Sensors*, 22(7):2726, 2022.
