# Supplementary material for: Improving prediction of cervical cancer using KNN imputer and multi-model ensemble learning
Source: PLoS One. 2024 Jan 3;19(1):e0295632. doi: 10.1371/journal.pone.0295632 (PMC10763959; doi:10.1371/journal.pone.0295632)
Supplement: Attachment — Submitted filename: Response_letter_Plos_One_Turki_Aljrees.pdf [file pone.0295632.s002.pdf]

Manuscript ID: PONE-D-23-24373

Article title: Improving Prediction of Cervical Cancer Using KNN Imputer and Multi-Model Ensemble Learning

November 23, 2023

Dear Academic Editor,

First of all, we would like to extend our sincere gratitude to Editor in Chief, Academic Editor, for allowing a re-submission of our manuscript, with an opportunity to address the reviewer's comments. We greatly appreciate the Reviewers' time spent reviewing the paper and assisting in its improvement. These comments are very helpful for revising and improving the paper, as well as the important guiding significance to the other research. We have studied the comments very carefully and made the corrections that we hope to meet with the final approval. We have done our best to modify the paper to reflect all the comments made by anonymous reviewers. These changes are fully addressed in this reply letter.

Below, we provide point-to-point responses explaining how we have addressed each of the Reviewer's comments. For clarity, our responses have been written in blue color. whenever we copy text from the manuscript, it is given in red color.

Academic Editor's Comments

Concern 1

Author has fulfilled the concerns, but I suggest to include more recent papers like "10.1007/s10462-023-10595-0" , "10.1016/j.imed.2022.07.002", "10.1038/s41598-023-40581-z".

Response: We are thankful for your insightful comment. Respected reviewer, We have added above mentioned works to the related work section of the paper.

Concern 2

Secondly, reconsider the placement of Figure 1 in the paper.
Also improve the figure captions to be more descriptive.
Please, incudes articles for critical research gap and future research
https://arxiv.org/abs/2212.02477
https://link.springer.com/article/10.1007/s11042-022-14061-x
https://doi.org/10.1016/j.eswa.2023.120477

Response: The authors are grateful for your valuable suggestions. We have added recently published works for cervical cancer survival prediction in the related work section. Also your other suggestions are incorporated.

References
